# Advanced Line-of-Sight (LOS) model for communicating devices in modern indoor environment

**DOI:** 10.1371/journal.pone.0305039

**Published:** 2024-07-05

**Authors:** Muhammad Waqas, Qamar Abbas, Ahsan Qureshi, Farhan Amin, Isabel de la Torre Díez, Carlos Uc Rios, Henry Fabian Gongora

**Affiliations:** 1 Department of Computer Science, Faculty of Computing and IT, International Islamic University, Islamabad, Pakistan; 2 Faculty of Computing and Information Technology, University of Jeddah, Khulais, Kingdom of Saudi Arabia; 3 School of Computer Science and Engineering, Yeungnam University, Gyeongsan, Republic of Korea; 4 Department of Signal Theory and Communications, University of Valladolid, Valladolid, Spain; 5 Universidad Europea del Atlántico, Santander, Spain; 6 Universidad Internacional Iberoamericana, Campeche, México; 7 Universidad Internacional Iberoamericana, Arecibo, Puerto Rico, United States of America; 8 Universidad de La Romana, La Romana, República; Government College University Lahore, PAKISTAN

## Abstract

The provision of Wireless Fidelity (Wi-Fi) service in an indoor environment is a crucial task and the decay in signal strength issues arises especially in indoor environments. The Line-of-Sight (LOS) is a path for signal propagation that commonly impedes innumerable indoor objects damage signals and also causes signal fading. In addition, the Signal decay (signal penetration), signal reflection, and long transmission distance between transceivers are the key concerns. The signals lose their power due to the existence of obstacles (path of signals) and hence destroy received signal strength (RSS) between different communicating nodes and ultimately cause loss of the packet. Thus, to solve this issue, herein we propose an advanced model to maximize the LOS in communicating nodes using a modern indoor environment. Our proposal comprised various components for instance signal enhancers, repeaters, reflectors,. these components are connected. The signal attenuation and calculation model comprises of power algorithm and hence it can quickly and efficiently find the walls and corridors as obstacles in an indoor environment. We compared our proposed model with state of the art model using Received Signal Strength (RSS) and Packet Delivery Ratio (PDR) (different scenario) and found that our proposed model is efficient. Our proposed model achieved high network throughput as compared to the state-of-the-art models.

## Introduction

Maturity in wireless communication made it an important means of communication during the recent couple of years. It is quite often that indoor environments are complex, suffer from dense obstacles, signal fluctuation occurs and they are characterized by non-line-of-sight (NLOS) [1]. Because of the indoor environment complexity, the accuracy of indoor localization is severely affected. Which results in a loss of received signal strength and leads to poor communication [2]. In today’s trend, a large number of communications come in an indoor environment, in which a user needs to get signals from the router that is placed away from him for communication purposes. However, this may result in communication channel high penetration loss, high shadowing effects, and obstacles that make the signals weaker and hence considerably degrade the efficiency of transmission [3]. It is a challenging task to provide wireless connectivity between every node operating in a complex propagation environment to achieve perfection in transmission and mitigate the low efficiency, latency, and ratio of low data rate among the nodes. Waves propagated considerably become weak by impeding through the walls, floor, ceiling, diffraction, absorption, and multiple reflections. Consequently, because of the absence of direct LOS, signals experience fading to a great extent [4]. Radio signals have less power to penetrate in comparison to other technologies such as GSM. So they are sometimes obstructed by moving obstacles such as humans and sometimes by static obstacles like walls, windows, doors, furniture, etc. These disruptions result in signal attenuation causing the radio signals to a low coverage area [5]. Inside the buildings, wireless local area networks (WLANs) frequently operate to provide connectivity to the user to perform data transmission and reception, but due to an excessive number of walls and other obstacles, the signal gets attenuate and the accuracy of the whole network is minimized [6]. With the wireless feature of communications, there is a raise in computing in every field. Smartphones, tablets, and laptops are now being used by every person even if they are at home or the office, so these devices become universal for communication and computing purposes [7]. Hence communication is needed to occur between multiple intended users. Thus demanding high throughput and high reliability [8]. The goals behind this are maximizing coverage and network boundaries while meeting certain constraints [9]. The advantage of indoor communication is that the Internet took place to help manpower, so the Internet nowadays is commonly used everywhere such as in educational institutes, industries, offices, etc. So each person is spending most of their time in the indoor environment even if he is at work or at home. The disadvantage in an indoor environment is that maintaining LOS with the router in an indoor complex environment is difficult while interacting with the internet. It is noticeable that many users are interacting with the internet in their daily lives such as in intelligent transport, industries, offices, hotels, universities, etc. [10]. Different kinds of internet are available and the most popular is wireless. In wireless internet service, there is no direct contact between the internet and the user of the internet. The signals are propagated and are spread everywhere. Some of which reached the destination and the rest are impeded due to the obstacles. Due to attenuation of signals the range of Wi-Fi routers is decrease and the performance of the network is reduced and there is a disturbance in the communication that is occurring or that is going to initiate. In the complex indoor environment, the signals need to reach the destination with the possibility of the direct LOS to enhance the accuracy of network and increasing the communication transmission range. The purpose of finding an optimal position is to aim to maximize the network throughput [11].

### Problem statement

In the existing wireless communication system to achieve improvement in the transmission and reception of data, various techniques and methods are introduced such as mobile robotic relay [14], placement of multiple access points [23], network partition [22], and many more ways. The most appreciable and important thing that is ignored and denied is the “optimal positioning” of access points (APs). Most of our daily data transmission occurs in indoor environments, such as Residential, Educational, Institutional, Industrial, Business, and many more. So signals decay and not being able to timely transmit data successfully because of penetration of signals in different obstructions (i.e. walls, windows, doors), sometimes signals reflect in the improper direction, and finally, due to long coverage transmission distance between transceivers, signals become weak. Due to the above-mentioned reasons radio signals lose their strength and become feeble and hence result in signals destruction.

### Preliminary investigation

**Fig 1:**
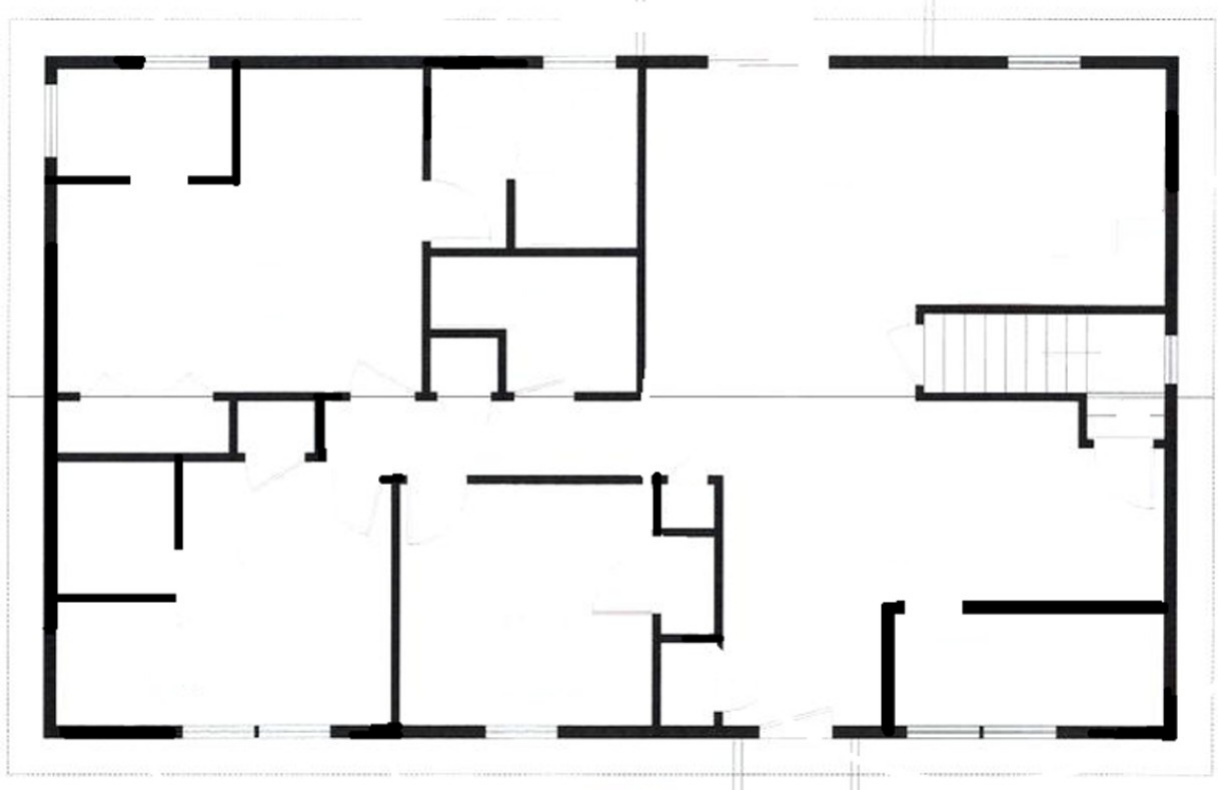
Modern building map.

In the preliminary investigation of the problem, Papers used different schemes to solve the problem of communication abilities between transceivers (sender and receiver) and improve packet delivery ratio (PDR), in the past data transmission in the indoor environment was not focused much. All of our business and dealings are to be done inside the buildings. That is why most of the communication occurs through an indoor environment through the office building. To transmit data in a regular and improved way is to maximize the range of signals and to maximize and improve LOS condition between transceivers all over the building, to ensure the reliability and integrity of the network. In the below Fig 1 map of the modern building can be seen with multiwall and complex indoor structure.

**Fig 2:**
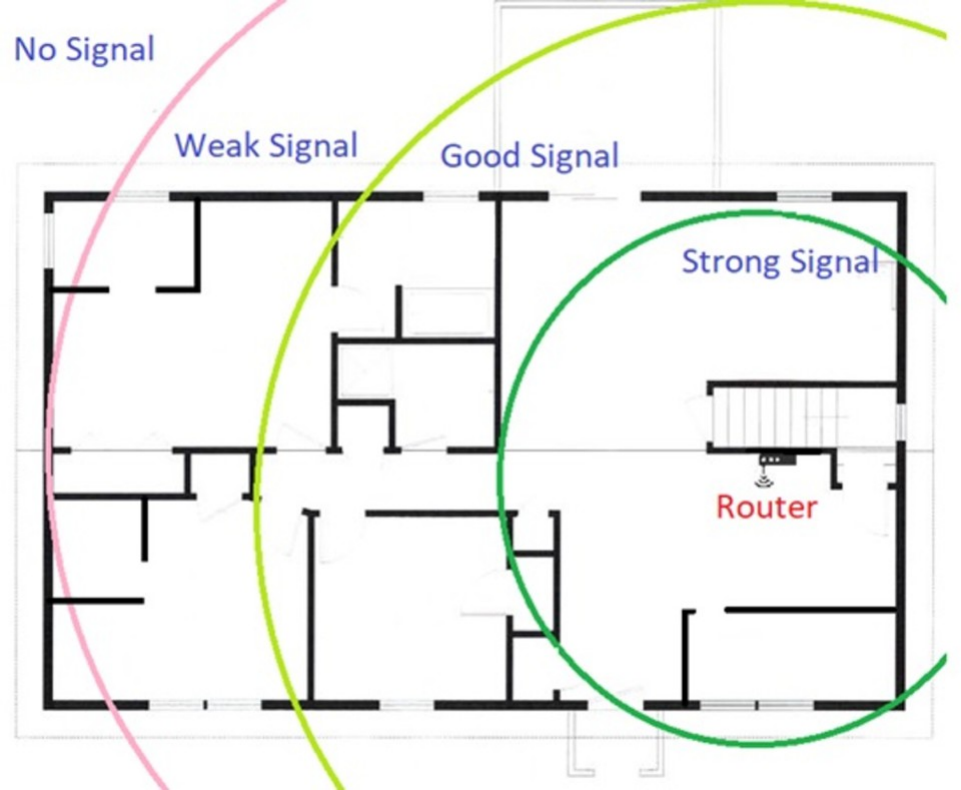
Modern complex building map with operating router.

In Fig 2, the router has to cover the whole volumetric area. There are a lot of areas to cover to provide strong signals for effective data transmission and reception while keeping many obstacles (walls, glass, doors) in mind. Finding and placing an optimal position for the router is a difficult task and little work has been done in the existing literature, in this regard. Many incidents can happen to signal their weakness. Signals face many obstacles when they propagate until reaching their destination.

**Fig 3:**
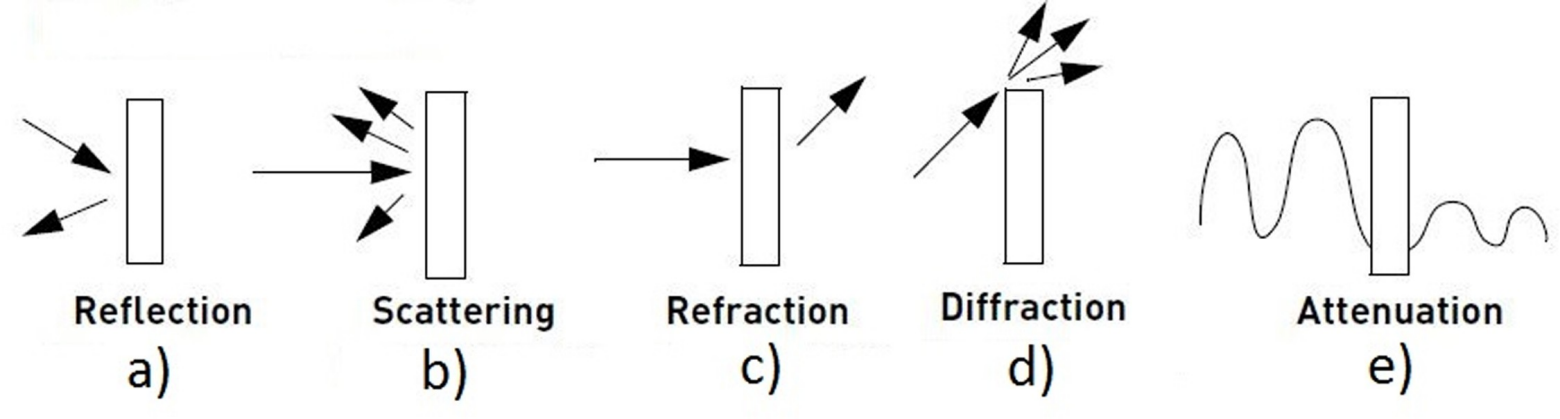
Range limiting factors.

Fig 3 indicates the range limiting factors inside the buildings. When signals propagate by the access point different phenomenon occurs to alternate the condition and direction of the radio signals. These above-mentioned factors and distance from access points (APs) to the dedicated devices cause the radio signals to become weak, by which signals are not able to transmit (carry) data in real-time, due to which packet deliver ratio (RDR) and throughput of network channel become unable to work and the whole network goes down.

- **Reflection:** Reflection is a phenomenon in which signals go back to the same medium that originates the signals after collision with obstacles (e.g. wall, glass window, door, cupboard). After collision radio signals inappropriately change their direction and hence the power of signals is lost and these become unable to carry data.
- **Scattering:** This phenomenon also happens when signals collide with some kind of obstacle and are divided into smaller parts and spread in different directions. This process leads to the signals in the situation of fading. Fading is known as the modification and the attenuation that affects radio signals over the propagation medium. The phenomenon of fading happens due to shadowing from radio obstacles that exist in the medium or multipath propagation.
- **Refraction:** Refraction is a phenomenon in which radio signals after passing through obstacles (e.g. wall, glass window, door, cupboard) bend inward or outward from a real angle. After collision radio signals inappropriately change their direction and hence the power of signals loses and these become unable to carry data.
- **Diffraction:** After the diffraction, the radio signals change their direction as they pass around the corner of the obstacle or through the aperture into the region of the geometrical shadow of the obstacle.
- **Attenuation:** Attenuation is the loss of strength of the signals in the network. Due to attenuation radio signals become distorted or indiscernible. Attenuation causes a low packet delivery ratio (PDR) and also reduces the reliability and availability of the whole communication system because NLOS (Non-Line-Of-Sight) limits signal strength in the indoor environment.

## Literature review

Previously, researchers were working to determine the optimal location of the network peripheral devices to enhance communication at the maximum level. To attain satisfactory results researcher uses different approaches and methods to solve the bottleneck. Usage of the Internet of Things (IoT) [12] has increased in real life due to its wide applications like e-health, automated control, and smart home. The composition of IoT is a group of interconnected (wirelessly) things (sensors, actuators, mobile devices, etc.) that communicate directly or indirectly furthermore, help one not satisfy a shared objective to achieve a common goal. For better communication and a larger network coverage area, it is better to place the Wi-Fi access point (AP) in the optimal position. To achieve optimal positioning, this paper focuses on how to position a Wi-Fi AP in a certain indoor setting. The suggested remedy offers a framework for an indoor map that provides a coordinate system and geographic representation. Where walls can be explicitly described, conveyed, and used to aid the AP placement process, such as point-by-point map data. The problem of optimization is tackled by the particle swarm optimization (PSO) algorithm and assessed by the k-nearest neighbors positioning algorithm. In the proposed method the walls are taken into consideration to keep in mind to avoid signal attenuation. Many drawbacks exist in the proposed solution such that the solution does not guarantee full coverage, it is not suitable for a small indoor environment (because placing APs close to each other may cause interference with each other) the wall identification process consumes a significant amount of processing time in the propagation model in the proposed optimization model as well. At present, the wireless mesh network (WMN) [13] is perhaps the most rising network, which offers low deployment cost. WMN is engaged with various applications like crisis circumstances, burrows, oil wells and combat zones, broadband home networking, and local area mesh networks. The optimal mesh route’s positions play an important and strongly influence the performance achievement of the WMN. To address the router node placement issue, this paper applies and modifies the electromagnetism-like mechanism (EM) met heuristic. To determine the ideal locations for mesh routers, it is assumed to take into account a population of arrangements encoded as particles subject to attractors and shocks as in electromagnetic frameworks based on Coulomb’s law. It mimics the attraction and repulsion of test guides placed in a holder to search for the best answer. It includes concurrently maximizing network availability and consumer inclusion. The outcomes show that the suggested method outperforms other methods in terms of client coverage and network connectivity. The proposed solution is computationally complex. Every communication requires stable connectivity [14] to enhance the transmission and receiving capability in applications where no communication infrastructures are available or they lack. So, there is a demand for long-lasting ad-hoc communication networks to permit the transmission of information. The approach to be successful is the wireless relays on mobile robots. Thus, enhancing the quality of transmission optimization of the whole network is necessary. To build an optimal communication network it must be needed to autonomously deploy relay robots to the proper position. An automatic Wi-Fi relay node positioning framework is developing an ad-hoc wireless sensor network to achieve a satisfactory dual-way communication level. To attain the desired goal a map of the operating environment is required to autonomously fully control the mobile robots. Robots relay devices must be positioned between the base station and the customer. To evaluate the sign intensity distribution of the mobile transfer robots and fixed base station, two Wi-Fi signal models were developed. To create the framework-based natural map, a visual laser-SLAM (visual synchronous localization and mapping) method was suggested. To locate the robot devices on the 2D natural map, the augmented Monte Carlo localization (AMCL) approach was used. To cover bigger territories different robots are utilized to advance the network. Every robot should have a different role (e.g. principal and subordinate robot). So availing the adequate performance of the network this approach is well. On the other hand, in the changing environment map needs to be built again and again so this process is computationally costly. Hundreds of useful applications [17] such as navigation, safety, healthcare, and entertainment are provided by vehicle-to-vehicle (V2V) infrastructure and vehicle-to-infrastructure (V2I) infrastructure in VANETs. Due to various radio impediments (static and dynamic) in the contemporary vehicular environment, communication signal strength decreases when vehicles obstruct the radio signals, and results in packet loss. Roadside units (RSUs) are used by VANETs for V2V and V2I communication. The current study focuses on the position of RSUs, where their positioning has an effective role in overall V2X (both V2V and V2I) communication. RSUs must be positioned so that the received signal strength (RSS) is appropriate for V2X communication and that there are fewer radio obstructions in the way of the signals. In metropolitan areas, modern complicated transportation infrastructure includes flyovers, interchanges, underpasses, irregular roadways, tunnels, dead-end streets, and curves. So, while taking into account road infrastructure, an ideal placement for RSUs, signal reflectors, and signal repeaters is provided. So the optimal positioning of Road Side Units (RSUs), reflectors, and repeaters is measured using geometrical values to improve maximizing and maintain Line-of-Sight LOS to improve packet delivery ratio (PDR) and make overall communication reliable. This method helps communication more accurately while keeping LOS between vehicles in modern complex road infrastructure. However, this approach only focuses on the implementation of the Internet of Vehicles (IOV). Vehicular Ad Hoc Networks (VANETs) [24] are used in a great number of real-world applications. The VANETs must be able to handle dynamic conditions. Modern transport infrastructures are complex interchanges, underpasses, road tunnels, and flyovers. Radio Propagation Models (RPMs) are greatly contrived by many factors such as complex road infrastructure units that are used while communicating by vehicles [25]. Radio propagation behavior is distinctively affected when they are conveyed in a restricted environment (such as in the tunnels) [26]. Different radio obstacles obstruct the radio signals in VANETs [27]. Radio signals operate at 5.9GHz frequency and roughly 5 cm wavelength; that is why they have relatively less power to penetrate [28]. In this paper, a radio propagation model is proposed which is new and computationally inexpensive and that is suitable for communication in road tunnels between vehicles [29]. In this formulated RPM, basic geometric values and properties of road tunnels are considered to acquire a link between geometric characteristics of tunnel and signal impede. Further signal attenuations that occur by obstacles that are moving inside the tunnel are assumed in the proposed RPM [30]. The benefit of the proposed RPM is that it does not overestimate the path loss in road tunnels, and predicted path loss is minimal from all other existing RPMs. Additional attenuation measurements make it computationally expensive. Also, the study only uses Wi-Fi nodes configured at 5 GHz for field measurements. The Internet of Things (IoT) is described as a system of correlated devices able to communicate with one another to complete particular useful tasks [31–33]. Routers working in a network ensure that these devices are connected [34]. It is difficult to optimize the placement of these routers in a distributed wireless sensor network (WSN) in a large structure. Software for computer-aided design (CAD) is viable since it offers a reliable and effective tool. To best place routers in a WSN, specialized organizations therefore rely on both; a helpful computer-aided design tool together with the expertise and flare of a sound master/specialist. This paper intends to build up another methodology dependent on the relationship between an effective Computer-aided design tool and a refined architect for the ideal positioning of routers in shrewd buildings for IoT applications. The philosophy follows a bit-by-bit system to weave an ideal network structure, having both automatic and designer intervention modes. Several contextual studies have been looked at, and the findings obtained demonstrate that the developed approach creates a synthesized network with the largest inclusion and the fewest routers in the overall network. In the proposed arrangement the entire network can get flawed because of the design’s absence of repetition to evade network disappointment that can be brought about by faulty nodes. The accurate position of the transmitter is vital to accomplishing productive, computerized, automated mining [35–37]. Tragically, the current algorithm for positioning generally yields low-exactness, inadequate materialness, and inconsistent last assessment because of the presence of NLOS error and because they disregard the position mistake of anchor nodes [38]. To connect this technology hole, they built up a novel localization technique by fusing adjustment, vibrational Bayesian unscented Kalman channel (VBUKF), complete least squares, and water cycling algorithm (WCA) to improve the positioning assessment exactness of ultra-wideband (UWB) placement system in a confounded underground structure. In the first place, the adjustment was carried out dependent on the choice of fitting reference nodes (RNs) in the LOS situations to appraise the scale-factor blunder, inclination, and placement mistakes for every AN; at that point, the VBUKF smoothing along with the thought of variation in time estimation clamor is utilized for lessen the impedance of NLOS attributable to the troublesome division of predisposition and NLOS blunder. Secondly, the TLS strategy is carried out for computing the objective position of the nodes dependent on the smoothed distances and adjusted places of ANs. In conclusion, the WCA installed conspire was executed to additionally improve assessment precision. The exploratory outcomes showed that the proposed TLS-WCA approach, after the execution of adjustment and VBUKF, was best ready to improve the localization exactness strikingly, and beat the other analyzed techniques, which can productively accomplish higher assessment precision, featuring remarkable localization execution. Path loss is a vital piece of signal fading as it is available altogether in remote sign transmission situations [39]. The ideal positioning of Access Point is of critical significance in building up an indoor WLAN arrangement that would uphold the entirety of its customers/hubs effectively. The reproduction in this paper attempts a creative way to deal with reenacting the path loss profile just as a got signal profile for any positioning of AP in a given organization, consequently empowering a method of picking an ideal situation of AP. By picking this ideal transmitter point, the general sign yield at the beneficiary point ought to be improved. The proposed WLAN Indoor Coverage model introduced here utilized an indoor environment that comprised chiefly of concrete walls. Take or glass impediments were not considered here yet they may handily be fitted into the model by adjusting the format and considering their particular attenuation factors which are promptly accessible. With this model, the client can foreordain the switch position to his/her necessities. Likewise, for the various switches, the condition can be adjusted decently without any problem. Although right switch information must be gathered. This proposed model can be executed much of the time and can have an effect in accomplishing the ideal yield signal that is more grounded, better, and quicker. Simultaneously, further alterations to the model are expected to change situations where the organization covers more than one story. For network-based indoor placement based on Wi-Fi access point (AP) radio signal strength estimations, a method is developed that incorporates channel modeling, positioning assessment, and error investigation methodologies [40]. A newly modest Bayesian learning approach is developed to model the radio force map (RPM) in the indoor environment, with RSS predictions substantially impacted by propagation attenuations, reflections due to multipath, and shadowing effects. An extra placement method with two stages is developed in light of the proposed RPM model. The position is determined in the initial phase for coarse positioning up to the indoor room scale. At that point, in the second phase for fine positioning, the RPMs for the specified indoor environment are utilized for position assessment in the environment with Bayesian estimation. The mean squared positioning inaccuracy is confirmed with the Bayesian Cramer-Rao lower bound. A broad investigation indicates that the normal placement inaccuracy of the proposed RPM-based methodology is 1.98 meters which accomplishes 22% upgrades through cutting-edge RSS-based indoor positioning techniques. Fundamentally, the proposed positioning and modeling strategy suitably takes advantage of the spatial relationship in the RSS tests to advance and improve positioning accuracy. Table 1 presents the summary of the state of the art in this research area. In this table, a reader can see the research along with the main features, pros, and cons given separately.

**Table 1:** Literature review summary.

Study	Main Feature	Strengths	Limitations
Xuan et al. [2017] [12]	• Optimization is implemented by the particle swarm optimization (PSO) • Evaluated by k-nearest neighbours placement algorithm	• The placement of AP is according to an indoor map that produces a coordinate system and geographic representation • The walls are taken into consideration for keep in mind to avoid signal attenuation	• The APs located close to each other cause destructive interference • In the optimization model, the wall identification process costs much of the computing duration period in the propagation model • Does not guarantee full coverage
Sayad et al. [2018] [13]	Electromagnetism algorithm	• Higher client coverage • Better network connectivity	Computationally complex
Lamri et al. [2017] [14]	Mobile robotic relay	• Dynamic communication ability • Stability in communication	• Computational costly in a changing environment • Expensive Setup
Chien et al. [2018] [15]	• Asynchronous particle swarm optimization (APSO) • Self-adaptive dynamic differential evolution (SADDE)	• Trying to reduce bit error rate (BER) with different-shaped antennas	• Inter symbol interference • Outage probability
Mozumdar et al. [2014] [16]	Robust and efficient synthesis algorithm	• Efficient communication • Location estimation	• In close wall proximity, if the base station is surrounded by a wall, then the algorithm processes much of the data to find out the router’s proper candidate positions.
Qureshi et al. [2013] [17]	Geometrical concepts are used to model and formulate the line-of-sight LOS	• Maintain LOS • Better Communication between V2X • Packet loss is minimized	• Implemented only in IOV
Md Tahmid et al. [2016] [18]	Python-based algorithm to calculate and obtain positional information	• Reduce the synchronization issues • Performance accuracy	• Select the wrong set of access points due to multipath • Integrity loss if the difference of coordination between the time of arrival and angle of arrival is more than three meters
Hongbo et al. [19] (2014)	Localization algorithm with peer phone assistance	• Synchronization strategy is used to avoid the possible signal collision between peer phones • Accuracy in location estimation	• Impact on battery performance due to assistance scanning • Possibility of inaccurate ranging result
Mariakakis et al. [2014] [20]	Employs geometric methods to place Wi-Fi Access Point	• Single access point installation • Real-time computations	• Inaccurate results if a physical turns missed
Puggelli et al. [2015] [21]	Two algorithms used for Building automation system • Mixed Integer Linear Program (MILP) • Polynomial Time Heuristic algorithm	• Consume power efficiently • The high value of throughput	• High running times for the synthesis of small networks (*∼*30 end devices) • Does not exactly provide the optimal solution
Zhong et al. [2015] [22]	Network partition scheduling with the help of two greedy algorithm	• WSN is alive all the time • Communication independency	• Reconnected WSN tolerates failure of router or gateway only once
Gunhak et al. [2015] [23]	Multiple access point installation	• Enhance network coverage area • Efficient Access point positioning in a multi-story building	• Cannot compute the whole volumetric coverage
Qureshi et al. [2016] [24]	Propagation Model	• Path loss is reduced • Do not overestimate the path loss	• Computational expensive • Measures path loss only at 5 GHz

It is evident from the current literature that, a power level of received signals in any communication system indicates its performance. In the indoor environment, the penetration of signals through different obstacles (e.g. walls, windows) reduces the coverage area of radio in the communication system and results in the establishment of radio links impossible to work efficiently. Many frameworks provide multiple access points, smart buildings, Mobile robotic relays, and network partitions which solve received signals power level issues. Maximizing and improving LOS is one of the major solutions to improve the packet delivery ratio (PDR) and as well to add availability to the overall communication network and enhance signal strength in an indoor environment. Signal blockage which results in signal attenuation is the main factor caused by the structure of modern buildings. Many of our business and social activities occur inside the buildings so it is a harsh challenge for signals to compete with obstruction and reach and spread inside the building to carry out our calculations and activities.

### Proposed optimal positioning model

In this section, we explain our proposed optimal positioning model. Our proposal comprised various components for instance signal enhancers, repeaters, reflectors etc. The geometrical concepts and rules are used to model and formulate LOS conditions. The purpose is to maximize and improve the LOS situation between the communicating devices and enhance the overall communication system’s integrity and reliability while also increasing the packet delivery ratio (PDR).

**Fig 4:**
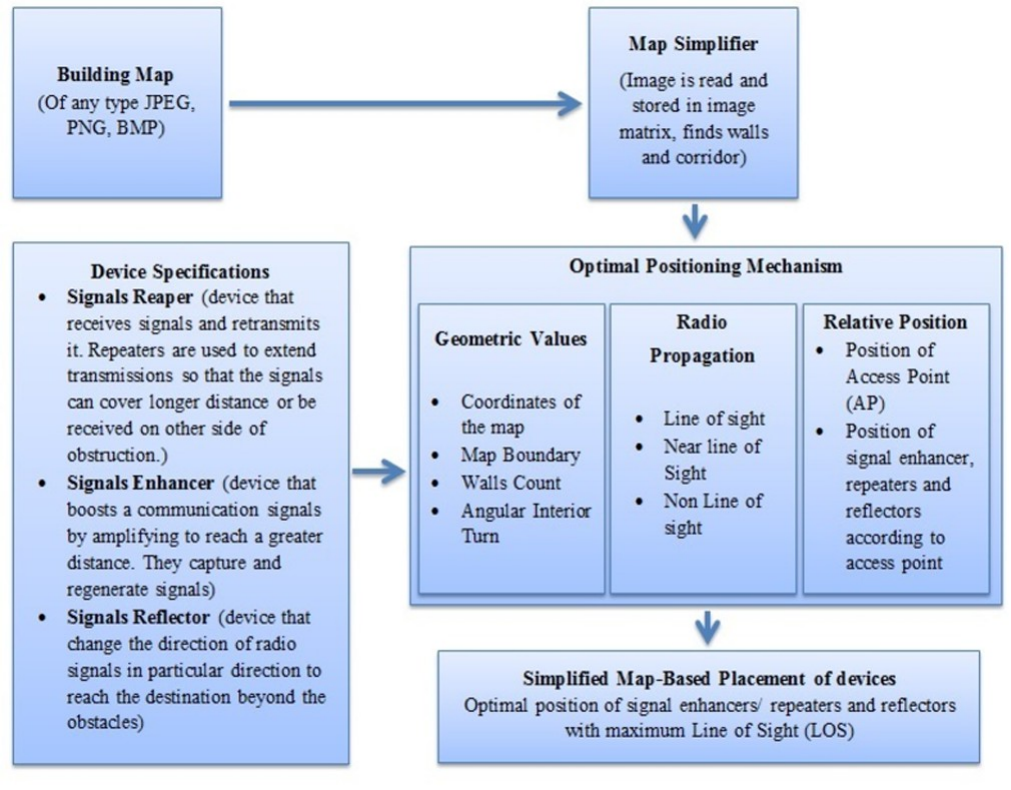
Components of our proposed model.

Fig 4 shows various components in our proposed model. Herein, every module is a separate entity that performs a specific activity.

**Fig 5:**
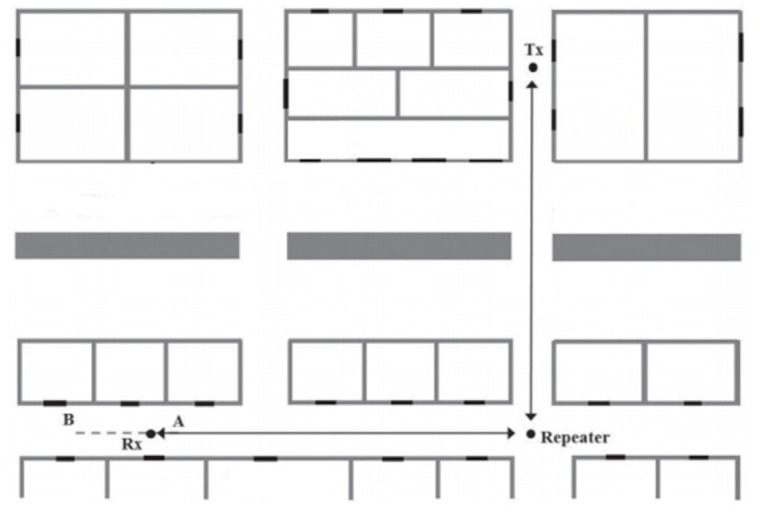
The positions of the transmitter, receiver, and repeater in our module.

The building nap module as shown in Fig 4 presents a Floor plan image of any type taken as the input of the candidate building. The proposed framework has to first take images of jpeg, png, BMP, etc. image. The Map Simplified Module shows the map of the candidate, the building is processed and stored in an image matrix. The outer boundary of the map is identified and the walls of the rooms are found also corridors are defined. Room positions are set after reading the map of the building. Measurement zones and building geometry are taken into consideration. Every pixel of the image is represented by the one matrix element integer from the set. The numeric values in the pixel are presented from 0 (black pixels) to 255(white pixels). The device specifications model show the different devices to cover the whole volumetric territory of the building. The devices help the signals to reach the destination within the building by reflecting and repeating the signals wave. Reflectors are the devices that modify the direction of the signals in the proper way to reach the destination beyond the obstacles according to desire. With the help of the repeaters, signals can overcome the problem of attenuation due to obstacle in their path that impedes them. Simply they collect signals and retransmit them. The main purpose of using repeaters is to extend the communication zone of the whole network. The strength of the radio signals is reduced as various radio obstructions in the indoor environment impede signals. So the signal enhancers are used to capture the signals and regenerate them. They boost the signals by amplifying them to cover the larger transmitter-receiver area. This is the core module in our proposed framework, which is positioning the router devices at the optimal position where the maximum communication radius will be maintained despite the presence of obstacles in the way of propagated radio signals. The position of the router (Access point) plays an important part in the efficiency of the communication system. The router is positioned in the indoor environment in such a manner that the received signal strength (RSS) of the router device by the end-user devices should be maximum and suitable for communication. The optimal position should be that from which overall volumetric dimensions of the operating area inside the building receive maximum signals. The router device should be placed in the best possible position for radio signal propagation so that barriers and other obstructions don’t significantly hinder the flow of signals. The optimal position of the router device is calculated by the number of steps. First of all geometrical values about the map are calculated, such as coordinates of the map found, the boundary wall of the map is identified and the corridor inside the map and the number of rooms are read out and stored in the image matrix form to generate a map by the program. All these values are received by the Map Simplifier module. Also if there is a curve inside the building it will also be taken into consideration and the optimal position for reflectors/repeaters calculated as in [17]. The main focus is to place the router device at such a place from where the LOS between communicating devices should be maximized. So optimal position of the router is such a position from where maximum signals reach out in the building, and the power of received signal strength is good in overall communication. It is difficult to maintain LOS conditions inside the building due to the number of obstacles in the path of signals (such as walls, cupboards, or cabins). So efforts are made for radio propagation to be Near the for every signal-receiving node. Nevertheless, those devices that are beyond the range of signals and are in a non- (NLOS) state may not be able to communicate properly due to signal decay. To overcome such an issue framework should place reflectors or repeaters at a reasonable distance to convey signals behind the obstacles (walls etc.) or at another bank of the building. Inside the building, there is an irregular pathway or corridor. These corridors are arranged at various angles from one another. In the corridor, the length of the corridor and the angle between the two corridors are key factors in LOS. The two devices are regarded as being in the LOS state if the angle difference between communication devices on distinct corridors is smaller than a threshold value with one another otherwise the devices in the various corridors are in the non-LOS condition so signal repeater/reflectors are needed to place to convey signal in such situation as in Fig 5.

**Fig 6:**
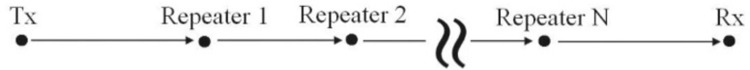
Radio repeaters on a straight-line path in our module.

Positioning of reflectors or repeaters depends upon relative position from the main router while positioning such assistance devices inside the building depends upon where a router device is placed while keeping in mind the received signal strength indication (RSSI). If there is a distance more than the threshold value (>25m) then a repeater device is needed to be placed in between to regenerate the signals for surety of signal strength as in Fig 6.

The result of the proposed model is that a simplified map-based placement of devices is performed to maximize and improve the LOS condition. The entire communication network’s accuracy increases the communication system’s availability and reliability while also enhancing and improving packet delivery ratio (PDR) while maximizing LOS and enhancing signal strength in an indoor environment.

### Algorithm 1: Our proposed Model.

**Begin**

   **//‐‐‐‐‐‐‐‐ Reading Map ‐‐‐‐‐‐‐‐//**

  Initial Phase

   Read-Image ()    // to find walls and corridor

   Convert x, y coordinates into pixel form store values in image matrix

  **//‐‐‐‐‐‐‐‐ Placing Router at Optimal Position ‐‐‐‐‐‐‐‐//**

  Input map coordinates and signal strength

  If signal-strength < threshold-signal-strength then

    If point value = 0 then {there is obstacle}

    Reiterate back position to first pixel that is not an obstacle

     Move another threshold1 back {that is new point}    //threshold1 for point

    Place point on map

  end if

  **//‐‐‐‐‐‐‐‐ Placing repeater or reflectors ‐‐‐‐‐‐‐‐//**

  Calculate signals strength at distance of threshold2 from router    // threshold2 for distance in meter

  If signal-strength < threshold-signal-strength then

  Place router {to enhance signals strength}

  Place point on map

  If found angle <90° then

  place reflectors

  place point on map

**End**

### Algorithm 2: Algorithm to find walls and corridor.

p–line segment that represent propagation path

W–set of line indicated walls of the map

j ‐ the index of wall

A[i]–angle of arrival matching to the wall

**//** ‐‐‐‐‐‐‐‐‐‐‐‐‐‐‐‐‐‐ **finding walls and corridor** ‐‐‐‐‐‐‐‐‐‐‐‐‐‐‐‐‐‐ **//**

 1. j = 0    // calculate the number of walls

 2. Mark all walls ‘W’ as unchecked    // traverse the set of walls

 3. **While** has uncheckedwall do

 4.  q ← NextUncheckedwall    // allocates next unchecked wall to q

 5.  v← Get point of intersection (p, q)    // to check whether line segment q

 6. intersects with p

 7. **If** v ≠ -1 then

 8.  A[j] ←v    // if q intersects with p, q is counted and stored in array

 9.  j←j+1

 10.  **end if**    //threshold1 for point

 11.  mark q as checked wall

 12.  **else**

       mark q as a corridor

 13. **end while**

### Signal attenuation and calculation model

To calculate the attenuation of the radio signals in complex environments by considering the distance between transceivers, attenuation exponent, frequency, and material that blocks the between transceiver devices. By using a general logarithmic function, we have

y=blog(x)+a (1)

As it is well defined that path loss and attenuation are directly proportional to γ,*f*,*h*_*diff*_. So, if, path loss is directly proportional we need a constant and we empirically identified that constant happened to be 2.

In Eq 1, we have two constants ‘a’ and ‘b’ which need to be tuned. Where ‘b’is the twice of product of γ,*f*,*h*_*diff*_ and *log* of *x*.


$$L_{RSS}(dBm)=2\gamma f\;h_{diff}log_{10}(x)-10avg_{mat}$$ (2)

*L*_*RSS*_ = Loss in received signal strength

γ = Attenuation through concrete

*f* = Frequency of Wi-Fi

*h*_*diff*_ = Height difference of the sender devices Tx and the Receiver devices Rx

*x* = Distance between sender devices Tx and Receiver devices Rx

*avg*_*mat*_ = Average attenuation of different construction materials

*x* is the distance between sender Tx and Receiver device Rx. That can be calculated by using

$$x=\sqrt{\left(x_{2}-x_{1}\right)+\left(y_{2}-y_{1}\right)}$$ (3)

This section describes the simple computationally inexpensive proposed model which is suitable and is used for calculating path loss in the corridor and the room in a modern indoor environment. This model is assessed based on an extensive field measurement campaign.

Our purpose is to propose such a model that uses a minimum set of parameters to measure the path loss in an acceptable domain. Two sorts of communication occur among the transceivers in an indoor environment. a) line-of-sight LOS communication and b) non-line-of-sight (NLOS) communication. The total path loss in LOS and NLOS scenarios inside the building is due to the existence of the barrier in the path of the signals. The path loss because of the presence of the obstacles is determined by utilizing the above-proposed formula. The above model (1) calculates the strength of the signals received inside the rooms as well as in the corridor in the indoor environment.

### Simulation results and detailed discussion

In this section, we discuss the achieved results in detail. First, we discuss our proposed evaluation criteria and then we discuss the achieved simulation results.

### Parameters for the experimentation

The assessment effects of choosing the optimal location for signal reflectors, repeaters, and enhancers in the indoor environment are elaborated on in this section. Simple signal reflectors can be used in the majority of modern indoor building environments to improve LOS conditions among communicating devices and improve the performance of two-way communication. This study not only reveals but also indicates all those conditions Because of this, basic signal reflectors can aid in maximizing LOS while also identifying the circumstances in which more cutting-edge devices like signal repeaters and signal enhancers should be employed. Simple reflectors are useful where there is a corner meaning loss of LOS in the next corridor so using simple reflectors just reflects signals in a particular direction. Signals lose their strength with increasing distance so using signal repeaters will be helpful for radio signals to enhance and regain their strength. The performance indicator and the parameters are shown in Table 2.

**Table 2:** Performance indicator.

Performance indicator	Unit	Typical Value Range
RSS	dBm	-110 to -10
PDR	Percentage	

We calculated the received signal strength (RSS) at the different locations inside the room and outside of the room in the corridor and its reliance on the distance between the communicating nodes in various multiple layouts.

### Experiment simulation and results

In this section, we presents simulation results and the key findings that were obtained during our research. We have measured RSS as a function and parameter of the distance between the communicating nodes. In addition, we have used the packet delivery ratio (PDR) as an additional performance metric to summarize the findings.

**Fig 7:**
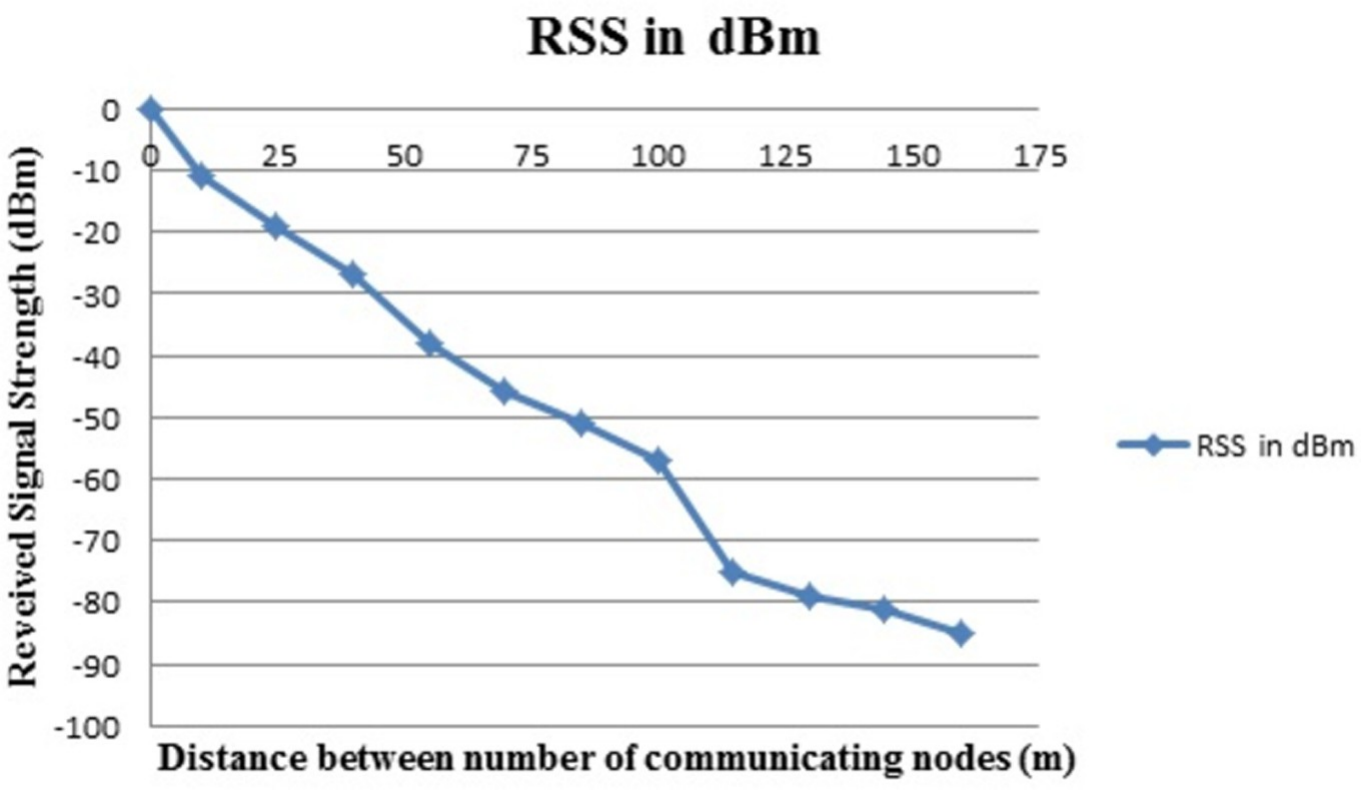
RSS in dBM.

Fig 7 shows the RSS in dBm. It is indicates that radio signals are good when the numbers of communication nodes are closer to each other and when these are in LOS.

Fig 7 shows the RSS in dBm and suddenly decreased after ‘Tr’ had moved a few meters away from Tx. It is evident that the radio signal rapidly degrades. When ‘Tx’ and ‘Tr’ are no longer in the LOS situation. After a 100m distance, there is a sudden fall in the strength of the signals due to NLOS due to presence of the obstacles in the path of the radio signals. When the distance between communicated nodes exceeds 125m, it is the region where signal quality deteriorates and causes a loss in packet delivery ratio. RSS increases as the distance between the sender and receiver decreases. The packet delivery ratio is a very crucial factor to measure the performance of any network. The total number of data packets arriving at destinations divided by the total number of data packets sent from sources will give you the packet delivery ratio. In other words, the packet delivery ratio is the proportion of packets delivered from the source to those received at the destination. When the packet delivery ratio is high, performance improves. Mathematically it can be shown as Eq (4).


$$\text{Packet}\;\text{Delivery}\;\text{Ratio}=\frac{\Sigma (\text{Total}\;\text{packets}\;\text{received}\;\text{by}\;\text{all}\;\text{destination}\;\text{nodes})}{\Sigma (\text{Total}\;\text{packets}\;\text{send}\;\text{by}\;\text{all}\;\text{source}\;\text{nodes})}$$ (4)

**Fig 8:**
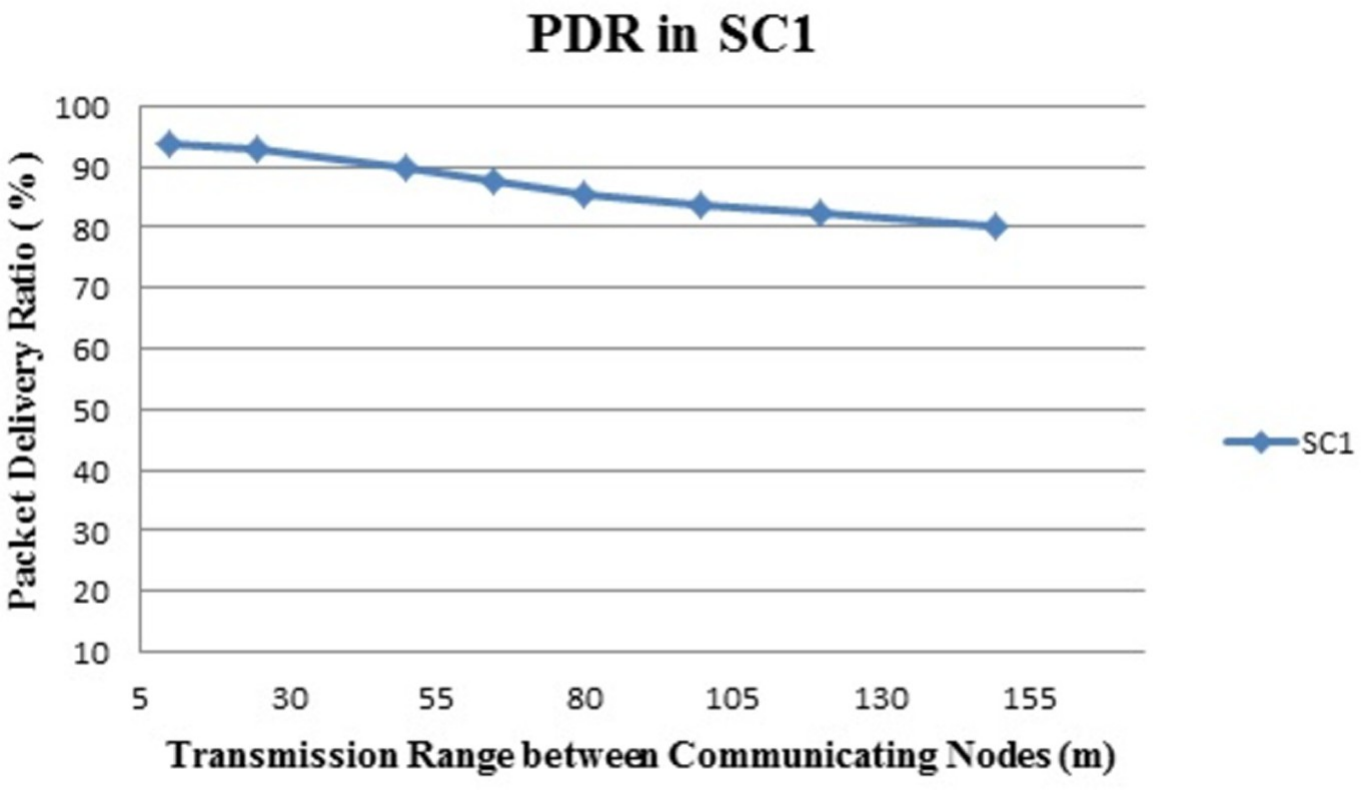
Scenario 1: Packet delivery ratio.

Fig 8 shows the Packet Delivery Ratio in percentage (%). The x-axis denotes the transmission range between communicating nodes in meters and the y-axis is PDR in %. It is clear from the above graph that, the packet delivery ratio of the proposed framework is very excellent. LOS situation obtained results are satisfactory in the NLOS situation too. The PDR result shows that delivering packets successfully is a challenge in an indoor environment.

**Fig 9:**
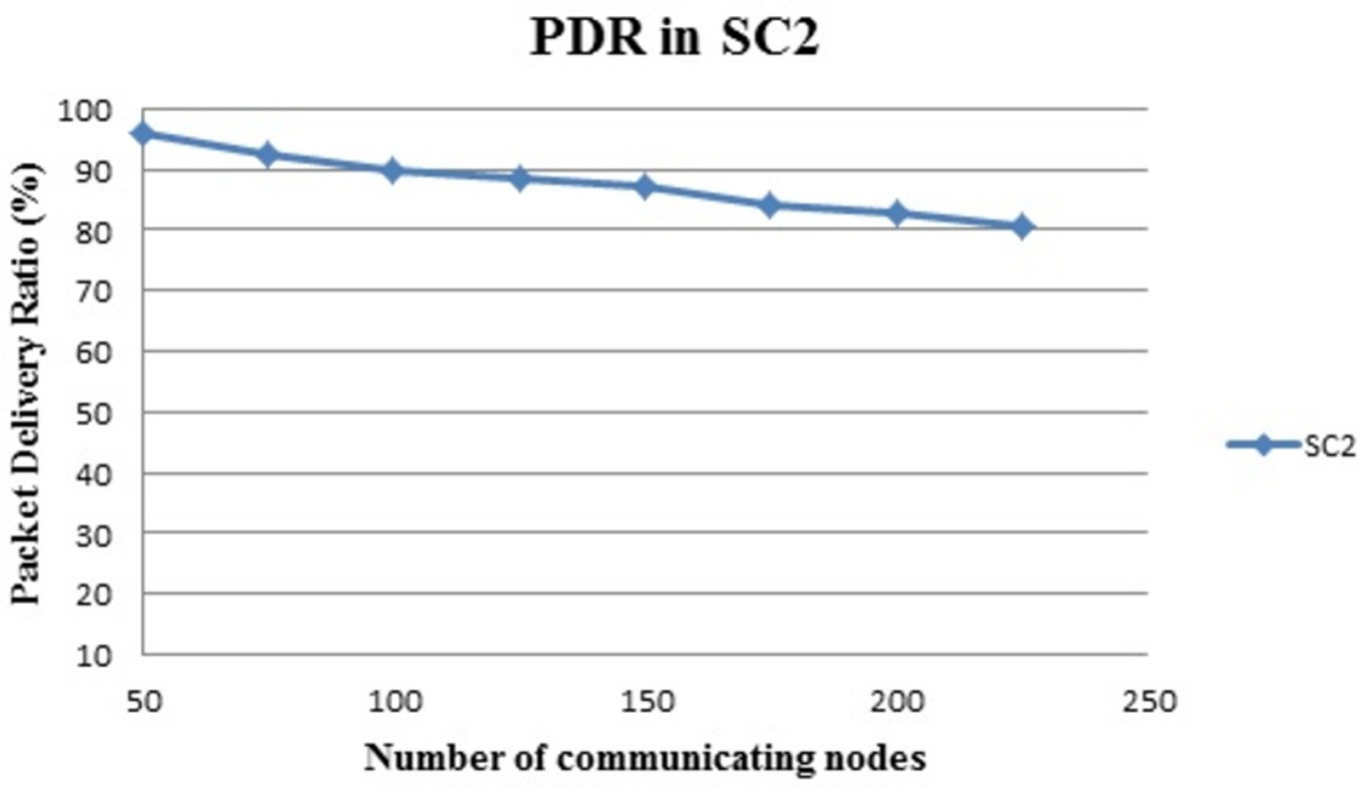
Scenario 2: Packet delivery ratio.

Fig 9 shows the PDR ratio in percentage (%). The x-axis denotes the ‘number of communication nodes’ and the y-axis is PDR in %. It is clear from the above graph that, the packet delivery ratio of the proposed framework is very excellent. In the indoor environment, where there is a LOS situation obtained results are satisfactory in the NLOS situation too. Communicating with large numbers of nodes while covering huge areas to deliver packets successfully is a challenge in the environment in the presence of obstructions.

### Comparison with state-of-the-art model

**Fig 10:**
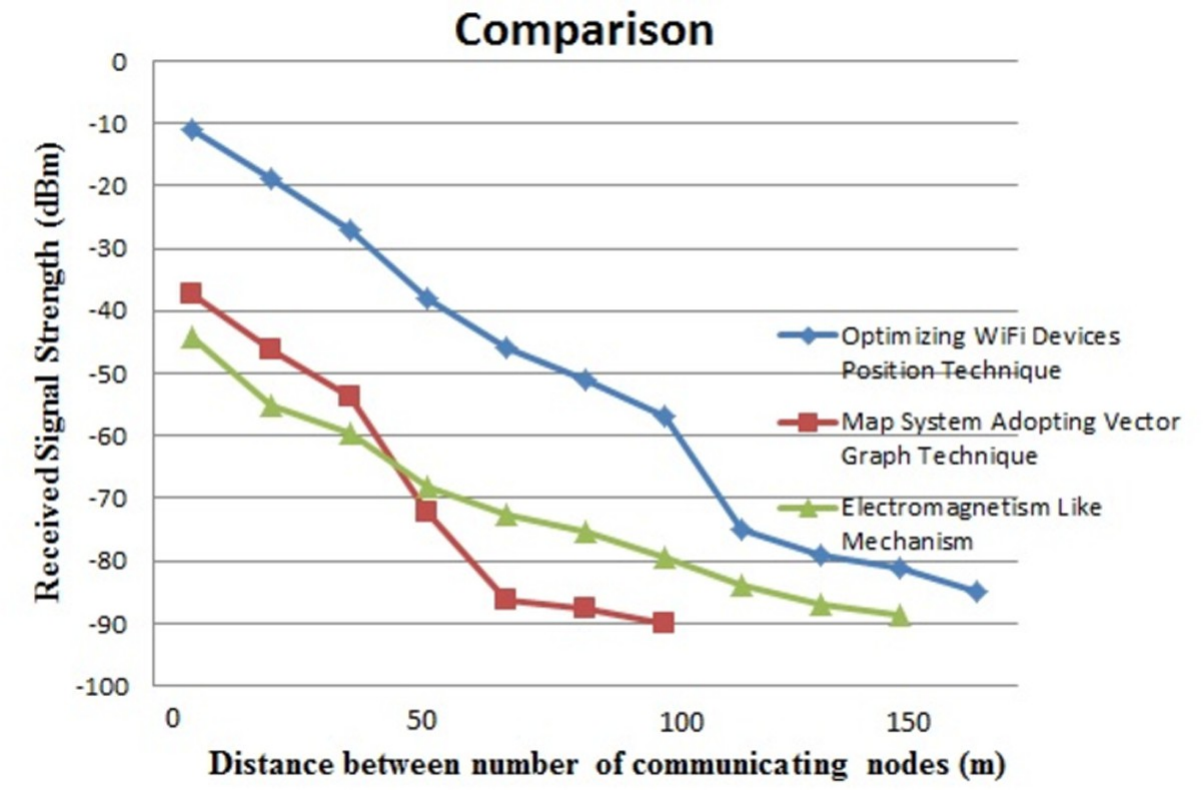
Comparison of RSS with state-of-the-art models.

In this section, we compared our proposed model with the state-of-the-art model. In this comparison, we choose the free-space path loss model as a state-of-the-art model. The following figure depicts a comparison between the free-space path loss model and our proposed method. In the following Fig 10 presents a comparison graph given below which describes the RSS of the proposed model.

As it is obvious from the comparison graph that the proposed approach shows encouraging results. it is evident from the results we can examine that the Overall Received Signal Strength (RSS) for the proposed model is satisfactory in both the LOS and non- (NLOS) layout of the router device. we have compared these results with another model named the free space model. The results show that our proposed model has optimal positioning of signal enhancers, repeaters, and reflectors in modern Indoor environments and shows better performance as compared to the existing model.

## Conclusion

Advancement in networks is coming up with numerous benefits in many fields. Internet nowadays is a world global remarkable development. Internet is accessed broadly in the indoor environment to perform business transactions, data sharing, and educational purposes. So it is always a challenge to enhance signal coverage area in a whole complex modern building. The proposed optimal positioning solution will play an important role in mitigating the issues related to the coverage area and accuracy of the whole network. So placing signal enhancers, repeaters, and reflectors at positions where their placement is accessible throughout every node is concerned with that particular network in a complex environment. This paper assesses the impact of formalizing the optimal positioning of Signal Enhancers, Repeaters, and Reflectors in the Modern Indoor Environment and presents helpful discoveries. Utilizing the basic signal reflectors in the most modern indoor environments gives a cost-effective solution, in maximizing and improving LOS between communicating devices or nodes to improve and boost communication. This research not only uncovers those circumstances wherein simple signal reflectors help amplify and improve LOS but additionally recognizes the situations where technologically advanced devices like signal enhancers and repeaters are to be used to add reliability to the overall communication system while maximizing LOS and enhance signal strength in an indoor environment. We believe that there is a lot of space in this domain, for improving the accuracy and reliability of networks operating inside buildings while maintaining, maximizing, and improving LOS. In the future, more techniques can be identified and combined with the proposed scheme. Also, the proposed scheme should be tested with frequency at 5GHz which may further enhance the efficiency of the communicating network, and the multi-story buildings are also taken into consideration.

## Supporting information

S1 Dataset (XLSX)
